# A germline exome analysis reveals harmful *POT1* variants in multiple myeloma patients and families

**DOI:** 10.1002/jha2.557

**Published:** 2022-09-02

**Authors:** Marja Hakkarainen, Jessica R. Koski, Caroline A. Heckman, Pekka Anttila, Raija Silvennoinen, Juha Lievonen, Outi Kilpivaara, Ulla Wartiovaara‐Kautto

**Affiliations:** ^1^ Applied Tumor Genomics Research Program, Faculty of Medicine University of Helsinki Helsinki Finland; ^2^ Department of Medical and Clinical Genetics/Medium, Faculty of Medicine University of Helsinki Helsinki Finland; ^3^ Department of Hematology Helsinki University Hospital Comprehensive Cancer Center, University of Helsinki Helsinki Finland; ^4^ Institute for Molecular Medicine Finland ‐ FIMM, HiLIFE ‐ Helsinki institute of Life Science University of Helsinki Helsinki Finland; ^5^ iCAN Digital Precision Cancer Medicine Flagship University of Helsinki Helsinki Finland; ^6^ HUS Diagnostic Center (Helsinki University Hospital), HUSLAB Laboratory of Genetics Helsinki Finland

**Keywords:** genetic analysis, multiple myeloma, germline mutations

## Abstract

Observations of inherited susceptibility to multiple myeloma have led to active research in defining predisposing genes to the disease. Here, we analysed 128 plasma cell dyscrasia patients’ germline whole‐exome sequencing data. Rare dominantly inherited pathogenic or likely pathogenic (P/LP) variant was found in 9.4% of the patients. Among the P/LP variants, *CHEK2 (*p. Thr410MetfsTer15) was the most prevalent (*n =* 5, 3.9%). Interestingly, P/LP variants in *POT1* were identified in three patients (2.3%). Our findings broaden the spectrum of *POT1*‐related cancers and demonstrate the importance of the germline genetic analysis in hematological malignancies.

## 1 SHORT REPORT

Epidemiological studies have identified family history of multiple myeloma (MM) as a risk factor for the disease already a century ago [1]. Prior whole exome sequencing (WES) studies in MM have **identified** potential rare germline variants in *DIS3*[2] and *LSD1/KDM1A* [3] **and also pointed out potential risk variants in** *ARID1A, USP45*[4], *EP300*[5], *DCHS1*, and *KIF1B* [6]. Additionally, genome‐wide association studies (GWAS) have recognized 25 different MM risk loci [7]. Our aim was to determine the frequency of rare pathogenic (P) or likely pathogenic (LP) germline variants and to discover novel genes linked to the MM predisposition in Finnish patients taking an advantage of the large population‐specific control set gnomAD noncancer Finns.

We recruited 128 unselected patients with MM and other plasma cell dyscrasias diagnosed and treated in the Helsinki University Hospital (HUH) district between April 2013 and October 2019. The study was approved by the HUH Ethics Committee. Written informed consent was obtained from all patients. Patient characteristics were retrieved from hospital records and the Finnish Hematological Registry (Table S1). The distribution of the patients’ clinical characteristics was consistent with the reported literature (Supplementary information). Germline DNA was extracted from skin biopsies collected using lidocaine with epinephrine local anaesthetic to avoid blood contamination. WES was performed to screen for single‐nucleotide variants and small insertions or deletions. A more detailed description of material and data acquisition, and analyses is presented in the Supplementary Information.

We designed an MM predisposition candidate gene list of 162 genes (Table S2). Genes were selected in accordance with the reports on germline predisposition (rare variants or GWAS hits) in MM/ monoclonal gammopathy of undetermined significance (MGUS), genes linked to the disease biology, or DNA repair (often mutated in germline predisposition to haematological malignancies). Also, genes frequently somatically mutated in MM/MGUS were included. We used a multistep filtering and strict criteria for the classification of the identified variants (Supplementary Information; Figure S1). Additionally, we compared the frequency of P/LP variants in the candidate list genes to the noncancer gnomAD global (*n* = 118 477) and gnomAD Finns whole exome subsets (*n =* 10 816) to distinguish between a true association and a chance. Finally, to identify potential novel predisposition genes we searched for P/LP variants in the COSMIC cancer census genes [8]. The criteria for further consideration as a novel predisposition gene were that at least two patients had a P/LP variant in the same gene.

**FIGURE 1:**
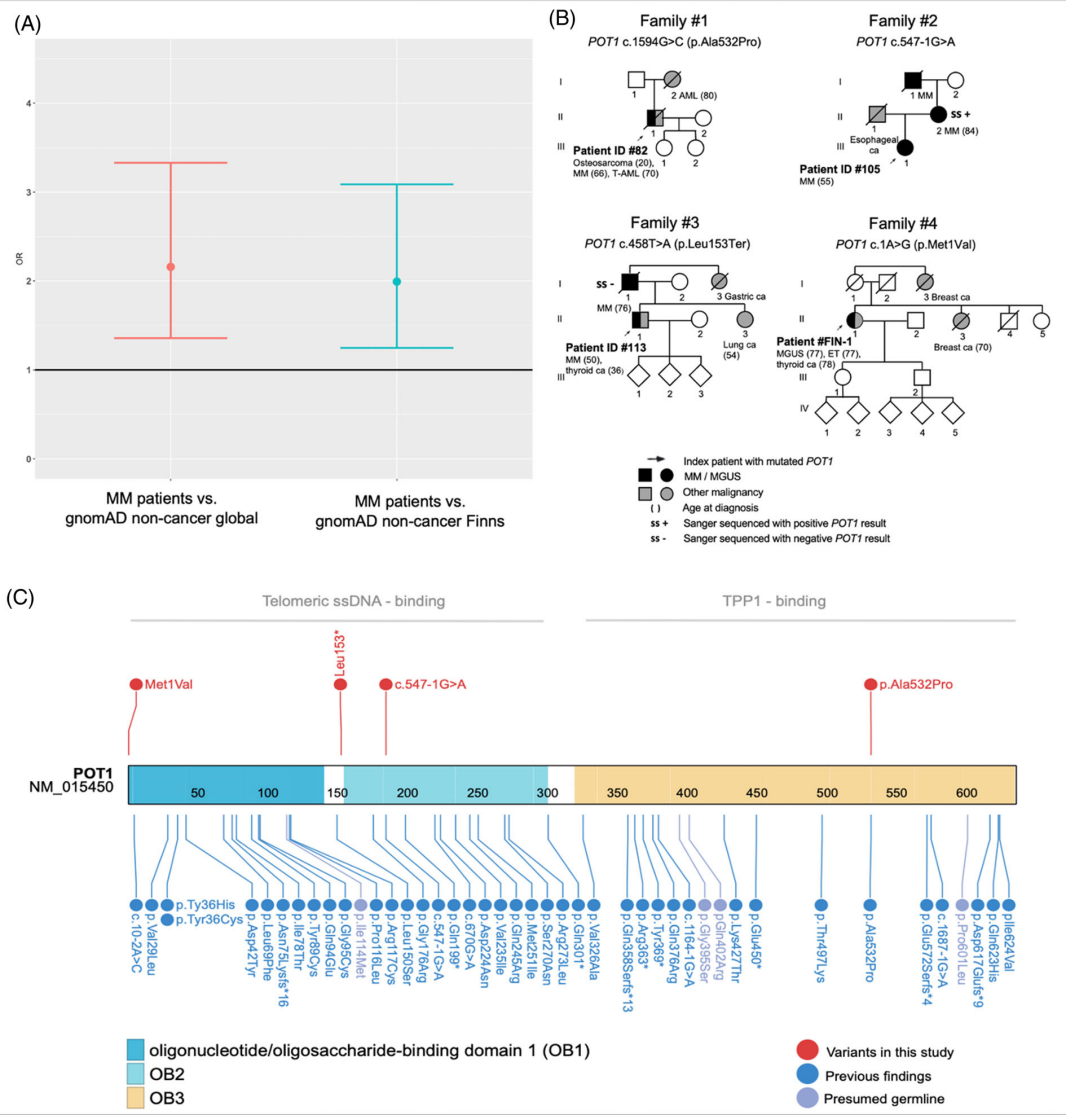
(A) Myeloma patients harbour significantly more germline pathogenic or likely pathogenic (P/LP) variants than gnomAD noncancer global and non‐cancer Finns (*p* = 0.0009 and *p* = 0.0035, respectively). (B) Pedigrees of the patients with germline P/LP POT1 variants. (C) Germline POT1 variants reported in this study and previously (AML [13], chronic lymphocytic leukaemia [14], chronic myelomonocytic leukaemia, hairy cell leukaemia, myeloproliferative neoplasms, myelodysplastic syndrome [15], Hodgkin lymphoma [16], angiosarcoma, breast sarcoma [17], colorectal cancer [18], gliomas [19], melanoma ([11], [20]), osteosarcoma [21], and thyroid cancer [22]). Abbreviatons: AML: acute myeloid leukaemia, ca: carcinoma, CI: confidence interval, ET: essential thrombocythemia, gnomAD: Genome Aggregation Database, MM: multiple myeloma, MGUS: monoclonal gammopathy of uncertain significance, OR: odds ratio, T‐AML: therapy‐related acute myeloid leukaemia.

A substantial proportion (12/128; 9.4%) of patients carried a heterozygous P/LP variant in dominantly (AD) inherited genes in their germline (Table 1). When including the heterozygous variants of recessively (AR) inherited genes, the number increased to 27 (21.1 %) (Table S3). The analysis aiming at identifying novel candidate genes for MM predisposition did not reveal genes that were not already included in the original candidate gene list. Furthermore, the study patients had significantly more P/LP variants in the candidate list genes compared to noncancer gnomAD *global* whole exome subset (OR 2.2, 95% CI 1.4–3.3, *p* = 0.0009, Figure 1A). To exclude a potential bias resulting from clustering of certain rare variants in Finland, we also compared the frequencies using the population‐matched gnomAD noncancer *Finns* and observed the same extent of enrichment (OR 2.0, 95% CI 1.3–3.1, *p =* 0.0035) (Figure 1A).

**TABLE 1:** Pathogenic and likely pathogenic variants of dominant inherited genes

**ID**	**Gene**	**Variant**	**OR (95% CI) compared to noncancer gnomAD global**	**OR 95% CI compared to noncancer gnomAD Finns**	**Coding impact**	**ACMG/AMP Classification (stratification criteria fulfilled)**	**Diagnosis**	**Age at diagnosis**	**Gender**	**Paraprotein subtype**	**Other primary malignancies**	**Family history ca or HD**
79	***ATM***	(NM_000051.4):c.7570G > C (p.Ala2524Pro)	154 (18–1289)	14 (2–118)	Missense	LP (PM1, PM2, PP5, PP2, PP3)	MM	54	F	IgG kappa	no	brother: cancer NOS
97	***ATR***	(NM_001184.4):c.516_529del (p.Val173IlefsTer7)	455 (41–5056)	N/A	Frameshift	P (PVS1, PM2, PP3)	MM	56	M	IgG lambda	no	no
40	***BRCA2***	(NM_000059.4):c.9118‐2A > G	N/A	N/A	Missense	P (PM1, PM2, PP3, PP5)	MM	64	F	IgG kappa	N/A	N/A
88	***BRCA2***	(NM_000059.4):c.8177A > G (p.Tyr2726Cys)	115 (14–928)	11 (1.3–85)	Missense	P (PVS1, PM2, PP5)	MM ‐ PCL	54	M	IgG kappa	no	no
53	***CHEK2***	(NM_001005735.2):c.1229delC (p.Thr410MetfsTer15)	10 (4–24)	2 (0.9–5.6)	Frameshift	P (PVS1, PM2, PP5)	MM	66	M	IgG kappa	prostate ca	N/A
66	***CHEK2***	(NM_001005735.2):c.1229delC (p.Thr410MetfsTer15)	10 (4–24)	2 (0.9–5.6)	Frameshift	P (PVS1, PM2, PP5)	MM	73	F	kappa light chain	no	N/A
78	***CHEK2***	(NM_001005735.2):c.1229delC (p.Thr410MetfsTer15)	10 (4–24)	2 (0.9–5.6)	Frameshift	P (PVS1, PM2, PP5)	MM	66	F	IgA lambda	basal‐cell ca	Father: gastric ca (died at 43), sister: breast ca, brother: prostate ca
91	***CHEK2***	(NM_001005735.2):c.1229delC (p.Thr410MetfsTer15)	10 (4–24)	2 (0.9–5.6)	Frameshift	P (PVS1, PM2, PP5)	sMM	61	F	IgA kappa	no	Mother's mother: leukaemia NOS
113	***CHEK2***	(NM_001005735.2):c.1229delC (p.Thr410MetfsTer15)	10 (4–24)	2 (0.9–5.6)	Frameshift	P (PVS1, PM2, PP5)	MM	50	M	IgA lambda	papillary thyroid ca (diagnosed at 36)	Father: MM (diagnosed at 76), sister: lung ca, paternal aunt: gastric ca
	***POT1***	(NM_015450.3):c.458T > A (p.Leu153Ter)	N/A	N/A	Nonsense	P (PVS1, PM2, PP3)						
82	***POT1***	(NM_015450.3):c.1594G > C (p.Ala532Pro)	913 (57–14686)	N/A	Missense	LP (PP3, PM2, PP5)	MM ‐ t‐MDS	66	M	IgG kappa	osteosarcoma, T‐MDS	Mother: AML
105	***POT1***	(NM_015450.3):c.547‐1G > A	N/A	N/A	Splicing	P (PVS1, PP5, PM2)	MM	55	F	kappa light chain	no	Mother: MM, mother's father: MM, father: esophageal ca
35	***PALB2***	(NM_024675.4):c.1592delT (p.Leu531CysfsTer30)	20 ([3]–147)	1.9 (0.3–14)	Frameshift	P (PVS1, PM2, PP3)	MM	65	F	IgG kappa	no	Father: gastric ca, sister: gastrointestinal ca (died at 41)

Abbreviations: ACMG/AMP, American College of Medical Genetics and Genomics/Association for Molecular Pathology  (stratification criteria fulfilled are in order of the strongest pathogenic criteria first), BP4: strong evidence of benign impact, ca: carcinoma, CI: confidence interval, gnomAD: Genome Aggreagation Database, HD: haematological disease, LP: likely pathogenic, MM: multiple myeloma, N/A: not assessed, NOS: not otherwise specified, OR: odds ratio, P: pathogenic, PCL: plasma cell leukaemia, PP2,3,5: supporting evidence of pathogenicity, PM1‐2: moderate evidence of pathogenicity, PS3: strong evidence of pathogenicity, PVS1: very strong evidence of pathogenicity, sMM: smouldering myeloma, t‐MDS: therapy‐related myelodysplastic syndrome.

The frameshift mutation *CHEK2* c.1229delC (p.Thr410MetfsTer15, also known as c.1100delC) was the most frequent variant we detected (5/129, 3.8%). The incidence of this variant in our study set is similar to familial breast cancer patients in Finland [9]. Although the frequency is significantly higher in our patients when compared to noncancer gnomAD global (OR 9.8, 95% CI 3.1–23.1, *p* = 0.0002), it is not substantial when compared to noncancer gnomAD Finns (OR 2.3, 95% CI 0.7–5.5, *p* = 0.0841). The increased risk for MM in our study seems to be akin as reported for breast cancer in a large pan‐cancer study from Denmark (HR 2.1, 95% CI 1.51–2.85, *p* < 0.001) [10]. However, we consider our finding of the high frequency of *CHEK2* c.1229deCl variant interesting. The lack of statistical significance in our study may relate to the limited number of patients, and the potential association of CHEK2 with MM remains to be validated.

Intriguingly, three patients (2.3%) carried an ultra‐rare P/LP germline variant in *POT1*[c.458T > A (p.Leu153Ter), c.1594G > C (p.Ala532Pro), or c.547‐1G > A] (Figure 1B,C). These P/LP variants identified in *POT1* are not specific to the Finnish population, since the minor allele frequencies (MAFs) are similar in Finland and globally (c.458T > A (p.Leu153Ter) MAF global: not available (N/A), Finland: N/A; c.1594G > C (p.Ala532Pro): MAF global: 0.0000319, Finland: N/A; c.547‐1G > A MAF global: N/A, Finland: N/A). Furthermore, c.1594G > C (p.Ala532Pro) has been described in a French family with cutaneous melanoma, and c.547‐1G > A in a Spanish patient with cardiac angiosarcoma ([11], [12]). The truncating variant c.458T > A (p.Leu153Ter) located in the telomeric single strand DNA binding domain of POT1 has not, to our knowledge, been reported before (Figure 1C).

POT1 binds to single‐stranded telomeric DNA, regulates telomerase activity, and suppresses response to DNA damage. Germline *POT1* mutations have previously been identified in various cancers: acute myeloid leukaemia (AML) [13], chronic lymphocytic leukaemia (CLL) [14], chronic myelomonocytic leukaemia, hairy cell leukaemia, myeloproliferative neoplasms (MPN), myelodysplastic syndrome [15], Hodgkin lymphoma [16], angiosarcoma, breast sarcoma [17], colorectal cancer [18], gliomas [19], melanoma ([11], [20]), osteosarcoma [21], and thyroid cancer [22]. Recognizing the diversity of hematological malignancies linked to germline *POT1* variants [15], we searched for P/LP *POT1* variants from additional samples in our germline WES database of 403 patients with haematological disorders *other than MM* (lymphoid neoplasms *n* = 76, myeloid neoplasms *n* = 280, immunological condition *n* = 31, mixed rare diagnoses *n* = 16). Here, we also identified one patient with a rare LP *POT1* variant c.1A > G (p.Met1Val) (Figure 1B,C, the pedigree of family 4, index #FIN‐1 [#4_II.1]). This variant distorts the initiation codon of *POT1* thus truncating the telomeric single strand DNA binding domain. The carrier patient had been diagnosed with essential thrombocythemia and papillary thyroid carcinoma. Surprisingly, further investigation of the patient's medical history revealed also MGUS.

We then examined the family history of the four patients identified with *POT1* variants. Two of the four families had MM in two and three generations, respectively (Figure 1B). In the family #2, we were able to confirm the *POT1* variant in the living family member (#2_II.2) with MM by Sanger sequencing. In the family #3, the index had had thyroid cancer preceding MM and carried P variants in *POT1*, *CHEK2*, and *MUTYH* but the father with MM (#3_I.1) shared only the *CHEK2* and *MUTYH* variants. All four *POT1* indices had a first‐degree family member with cancer. Furthermore, three of them had also been diagnosed with another *POT1‐*related malignancy: AML, MPN, osteosarcoma, and two cases of thyroid cancer (Figure 1B) ([13], [15], [21], [22]). The median age of MM/MGUS onset in the *POT1* carriers was 60.5 years old (range 50–77 years), which is similar to the onset of MM in general. Estimation of penetrance of the *POT1* variants regarding MM predisposition calls for larger study series.

POT1 functions in telomere maintenance. In familial melanoma patients and in one AML patient with germline *POT1* variants telomere length was reported to be increased ([11], [21]), whereas in CLL cases telomere length was normal [14]. Unfortunately, none of our *POT1* germline variant carriers were fit for telomere measurements: two were older than the reference age of 80 years, one had undergone allogeneic haematopoietic stem cell transplantation (HSCT), and one withdrew from further investigations.

Our findings support the role of germline genetics in myeloma; one tenth of the unselected study patients carried rare and harmful variants in genes associated with MM/MGUS, or DNA repair. Most interestingly, we discovered four cases and one first‐degree relative with P/LP *POT1* variants and plasma cell dyscrasia. Rare *POT1* variants have not previously been associated with MM predisposition. However, a GWAS study by Went et al. [23] suggested that *POT1* may be linked to MM. Our findings solidate this finding and assign MM as a novel cancer included in the spectrum of POT1‐associated malignancies.

In conclusion, our findings strengthen the connection between inherited genetic factors and plasma cell dyscrasia. We hereby suggest considering germline *POT1* testing for MM patients, especially if the patient or the family history presents with a *POT1* spectrum of solid or haematological malignancies. Allogeneic HSCT is an option only for some younger MM patients, and clinicians should carefully evaluate the need for germline testing when considering a sibling donor. While MM remains incurable, the awareness of the MM patients’ risk for other *POT1‐*associated malignancies can advance the early detection of these cancers and their potential cure.

## AUTHOR CONTRIBUTIONS

OK and UW‐K designed the study. MH collected the clinical data, designed the candidate gene list, analyzed the results and the exome data, and drafted the manuscript together with OK and UW‐K. JRK conducted the comparison between the study patients and population‐matched gnomAD subsets and contributed to the statistical analyses, figures, and the manuscript. CAH organized the sample collection and exome sequencing at FIMM. PA, RS, and JL collected samples for the study. All authors have read and approved the manuscript

## CONFLICT OF INTEREST

The authors declare no competing financial interests. CAH received research funding from Celgene, Kronos Bio, Novartis, Oncopeptides, and Orion Pharma unrelated to this study.

## Supporting information



Supplementary material

## Data Availability

WES datasets have not been deposited in a public repository due to privacy and ethical restrictions but are available upon reasonable request. The study was approved by the HUH Ethics Committee. Written informed consent was obtained from all patients.
