# Supplementary material for: A germline exome analysis reveals harmful POT1 variants in multiple myeloma patients and families
Source: EJHaem. 2022 Sep 2;3(4):1352–7. doi: 10.1002/jha2.557 (PMC9713058; doi:10.1002/jha2.557)
Supplement: Supplementary file 1 — Supplementary material [file JHA2-3-1352-s001.docx]

Supplementary Information

**A germline exome analysis reveals harmful *POT1* variants in multiple myeloma patients and families**

Marja Hakkarainen^1, 2, 3^, Jessica R. Koski^1, 2^, Caroline A. Heckman^4, 5^, Pekka Anttila^3^, Raija Silvennoinen^3^, Juha Lievonen^3^, Outi Kilpivaara^1, 2, 5, 6^ *, Ulla Wartiovaara-Kautto^1, 3^ *

^1^ Applied Tumor Genomics Research Program, Faculty of Medicine, The University of Helsinki

^2^ Department of Medical and Clinical Genetics / Medium, Faculty of Medicine, The University of Helsinki

^3^ Department of Hematology, Helsinki University Hospital Comprehensive Cancer Center, The University of Helsinki

^4^ Institute for Molecular Medicine Finland - FIMM, HiLIFE - Helsinki institute of Life Science, The University of Helsinki, Helsinki, Finland

^5^ iCAN Digital Precision Cancer Medicine Flagship, University of Helsinki, Finland

^6^HUS diagnostic center (Helsinki University Hospital), HUSLAB Laboratory of Genetics

*These authors contributed equally to this work

Corresponding authors:

Ulla Wartiovaara-Kautto, MD, Ph.D. [ulla.wartiovaara-kautto@hus.fi](mailto:ulla.wartiovaara-kautto@hus.fi)

Outi Kilpivaara, Ph.D. [outi.kilpivaara@helsinki.fi](mailto:outi.kilpivaara@helsinki.fi)

Supplementary material and methods

1. Sample preparation and whole-exome sequencing
2. Variant analysis and annotation strategies
3. Statistical analysis
4. Patient series
   1. Statistical comparison

Supplementary figures and tables

Supplementary figure 1: Study workflow

Supplementary figure 2: Kaplan-Meier overall survival curves

Supplementary table 1: Patient characteristics

Supplementary table 2: The candidate gene list

Supplementary table 3: Pathogenic and likely pathogenic variants of dominant and recessively inherited genes

Supplementary references

1. **Sample preparation and whole-exome sequencing**

DNA was extracted from fresh skin biopsies using the DNeasy Blood and Tissue kit according to the manufacturer’s directions (Qiagen, Hilden, Germany). The Institute for Molecular Medicine Finland (FIMM, Helsinki, Finland) conducted the whole-exome sequencing (WES) from the biopsies as described previously (1) using kits provided by Agilent Clinical Research Exome (Agilent, Santa Clara, CA, USA), Nimblegen SeqCap EZ exome v2.0 (Roche, Basel, Switzerland) or The SeqCap EZ MedExome (Roche, Basel, Switzerland). The reported variants were either confirmed from somatic WES originating from bone marrow-derived CD138+ plasma cells, or in peripheral blood mononuclear cells by Sanger sequencing (*n =* 2). Sanger sequencing was performed in our laboratory from mononuclear peripheral blood samples. Primer protocols and sequences are available upon request.

1. **Variant analysis and annotations strategies**

The variants in the WES data were analysed with the analysis and visualization tool BasePlayer (2).

Only non-synonymous and splice site variants with a minor allele frequency (MAF) of <0.01 in the whole Genome Aggregation Database (gnomAD; version 2.1;) (3) non-cancer and non-cancer Finns subsets were filtered further. Poor-quality variants were filtered out using quality measures (allelic fraction >20%, coverage >6, genotype quality >20, and QUAL >20), and a 1000 genomes mappability pilot mask track (Figure S1). Insertions and deletions were retained for further analysis. The following algorithms evaluated missense variants: 1) CADD (score >15), 2) DANN (score >0.93), 3) Revel (score >0.5), with an inclusion cut-off of at least two pathogenic predictions. Variants that passed the filtering steps were classified according to the American College of Medical Genetics (ACMG) classification rules in Varsome (version 11.2) (4). For variants classified as pathogenic or likely pathogenic (P/LP), a literature review was performed. The second approach was applied to find novel predisposing genes by identifying P/LP variants in the same gene in at least two patients. Genes were filtered by COSMIC cancer census genes (5) and the variants were analysed following the same steps as in the candidate-gene analysis (Figure S1). All reported P/LP variants were confirmed manually by visual inspection of the *bam* file in BasePlayer.

1. **Statistical analysis**

Statistical analyses were performed using SPSS version 27.0 Software (IBM, Endicott, New York, USA), and R (version 4.1.0) (6). Fisher’s Exact test was used to assess the enrichment of binary features. Mann-Whitney-U test was performed to compare differences in means or medians for age. All tests were two-sided. The differences were considered significant if *p* < 0.05. The Kaplan-Meier curves were calculated to estimate overall survival and time-to-last-control distribution.

1. **Patient series**

Written informed consent was obtained from all patients. ​​The median age for the whole patient series was 63 years and the average age was 62 years (range 26 – 82).

**4.1 Statistical comparison**

Age at diagnosis; gender

No statistical significancy was found when we compared the distribution of age at diagnosis and gender between the groups of patients with and without P/LP germline variants (*p* = 0.2; 0.19).

Multiple myeloma subtypes and risk stratification

There were no significant differences in the heavy- and light chain secretion (IgA kappa, IgA lambda, IgD kappa, IgD lambda, IgG kappa, IgG lambda, kappa light chain, lambda light, non-secretory) between the two groups (*p* = 0.16 to 1).  For the international staging system (ISS) the median score was two in both groups and the distribution of the scores did not show significant differences (*p* = 0.13 to 0.8). Between the patients with and without P/LP variants, there were no significant differences in the international myeloma working group risk (IMWG) stratification scores (*p* = 0.16 to 1).

Family history

A self-reported family history of malignancy was available from 91/128 (71 %) patients. Positive family history was defined as at least one 1st degree or 2nd degree family member with a history of any cancer. Family history was positive in 50/128 (39 %) of the cases, of which 12/128 (9.4%) were haematological malignancies. No significant differences in the family history of any cancer were detected when we compared the patients with and without P/LP variants (*p* = 0.52). However, patients with P/LP variants in genes with dominant inheritance had more often positive family history of haematological malignancies compared to rest of the patient group (4/12; 33.3% vs 9/116; 7.8%, *p* = 0.02).

Second primary malignancy

Second primary malignancy was diagnosed with 6/27 patients (22.2%) with germline P/LP variants versus 17/101 patients (16.8%) without recognized P/LP variants (p = 0.57). Information about the second primary malignancy was missing from 17 patients (13.3%).

Survival

Follow-up time and disease status were available from 123 patients (96.1%). The median follow-up time was 65 months (range 0 - 203 months). At the end of the follow-up time, 70 patients (54.6%) were deceased. There were no statistically significant differences between the patient groups in the Kaplan-Meier survival analysis (Log Rank *p* = 0.3; Figure S2).

**Supplementary figure 1: Study workflow**


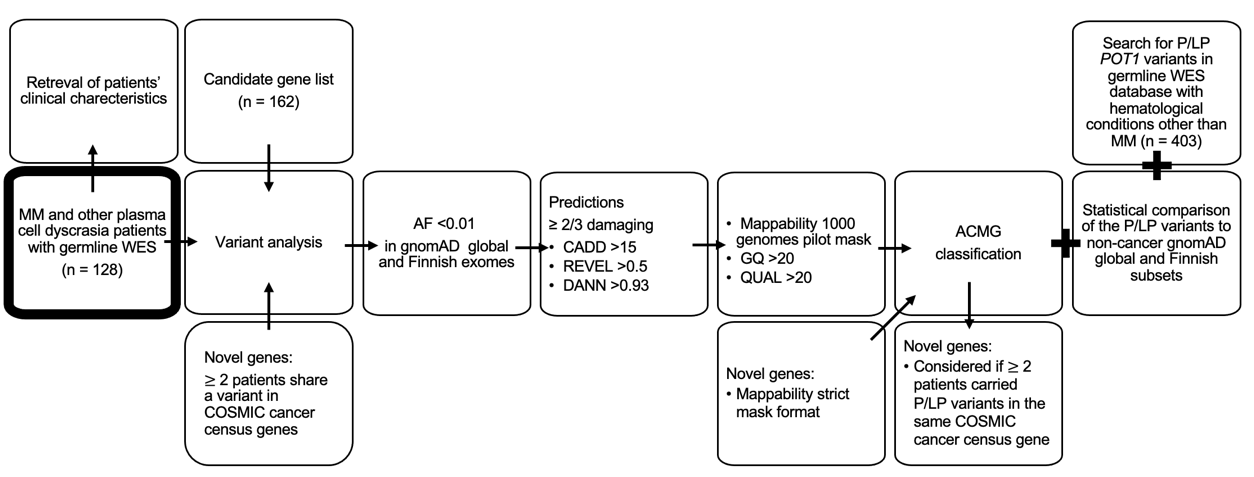
Abbreviations: MM: multiple myeloma, WES: whole-exome sequencing, AF: allele frequency, gnomAD: Genome Aggregation Database, CADD: Combined Annotation Dependent Depletion, REVEL: rare exome variant ensemble learner, DANN: deleterious annotation of genetic variants using neural networks, GQ: genotype quality, QUAL: quality, ACMG: American College of Medical Genetics

**Supplementary figure 2: Kaplan-Meier overall survival curves**


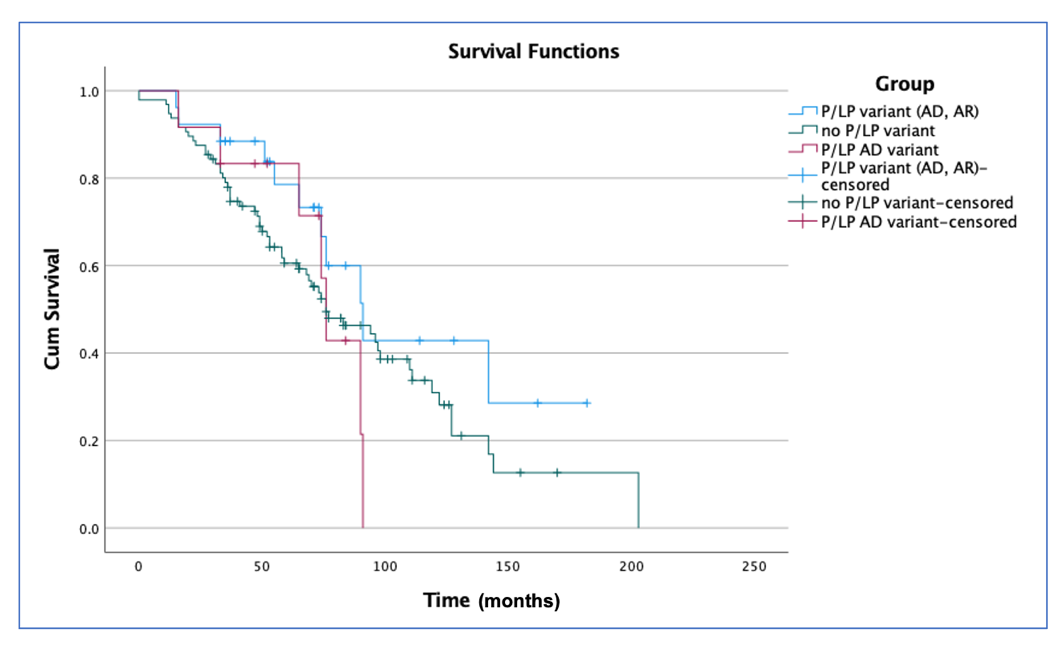
Patients were divided into three groups: 1. patients with germline pathogenic or likely pathogenic (P/LP) variant with dominant or recessive inheritance [P/LP variant (AD, AR)], 2. patients with no germline P/LP variants [no P/LP variant], 3. patients with dominant inheritance P/LP variants [P/LP AD variant]. No statistically significant difference was found between the groups

**Supplementary table 1: Patient characteristics**

| **Patient ID** | **Gender** | **Diagnosis** | **Age at diagnosis** | **Paraprotein subtype** | **ISS score** | **IMWG risk stratification** | **CRAB criteria** | **Other primary malignancies** | **Family history of cancer (CA) or haematological disease** | **Allo-HSCT (yes/no)** | **P / LP variant** |
| --- | --- | --- | --- | --- | --- | --- | --- | --- | --- | --- | --- |
| 1 | F | MM | 62 | IgA lambda | 3 | standard | A | no | N/A | no |  |
| 2 | F | MM | 62 | IgG kappa | 2 | high | AB | no | daughter's son: rhabdomyosarcoma | no |  |
| 3 | M | MM | 72 | IgA kappa | 2 | standard | AB | no | mother: breast ca, brother: gastric ca | no |  |
| 4 | F | MM | 63 | IgG kappa | 1 | standard | A | no | no | no |  |
| 5 | M | MM | 66 | IgG lambda | 1 | standard | AB | no | no | no | *FANCM* c.5101C>T (p.Gln1701Ter) |
| 6 | F | MM | 63 | lambda light chain | 2 | standard | AB | breast ca | paternal grandmother mother: breast ca | no |  |
| 7 | M | MM | 74 | non secretory | 2 | standard | A | no | no | no |  |
| 8 | F | MM | 64 | IgA kappa | 3 | standard | CAB | no | no | no |  |
| 9 | F | MM | 65 | IgA kappa | 1 | standard | B | papillary thyroidal ca | no | no |  |
| 10 | M | MM | 62 | kappa light chain | 1 | standard | A | no | sister: breast ca | no |  |
| 11 | F | MM | 57 | lambda light chain | 1 | standard | AB | no | no | yes |  |
| 12 | M | MM | 49 | IgD lambda | 1 | standard | B | t-MDS | no | yes |  |
| 13 | M | MM | 62 | IgA lambda | 2 | high | A | no | no | no |  |
| 14 | M | MM | 57 | lambda light chain | 1 | standard | B | no | mother: ovarian ca | no |  |
| 15 | M | MM | 70 | IgG kappa | N/A | N/A | 0 | no | no | no |  |
| 16 | F | MM | 59 | IgA lambda | 1 | standard | 0 | no | identic twin sister: CLL | no |  |
| 17 | M | MM | 65 | IgG kappa | 2 | standard | 0 | no | no | no |  |
| 18 | F | MM | 56 | lambda light chain | 2 | standard | B | breast ca | two sisters: breast ca | yes | *RAG1* c.2615delT (p.Leu872Ter) |
| 19 | M | MM | 66 | IgA lambda | 3 | standard | RAB | no | no | no |  |
| 20 | M | MM | 50 | IgG kappa | 1 | standard | B | no | father: liver ca | no | *MUTYH* c.536A>G (p.Tyr179Cys) |
| 21 | M | MM | 45 | IgG kappa | N/A | N/A | AB | no | no | yes |  |
| 22 | M | MM - PCL | 56 | IgG kappa | 2 | standard | B | basal cell ca | no | no |  |
| 23 | M | MM | 61 | kappa light chain | 1 | standard | B | no | paternal aunt: colon ca | no |  |
| 24 | F | MM- PCL | 69 | IgG kappa | 3 | standard | A | no | no | no | *FANCI* c.286G>A (p.Glu96Lys) |
| 25 | M | sMM - AL-amyloidosis | 58 | IgG kappa | 2 | standard | 0 | no | no | no |  |
| 26 | M | MM | 68 | IgA lambda | 3 | high | AB | no | no | no |  |
| 27 | M | MM | 75 | IgG kappa | 2 | standard | A | LGL-leukaemia | no | no |  |
| 28 | M | MM- AL-amyloidosis | 63 | kappa light chain | 1 | standard | B | no | son: DLBCL, father colon ca | no |  |
| 29 | M | MM - PCL | 68 | IgA lambda | 2 | standard | B | no | no | no |  |
| 30 | F | MM | 65 | IgG kappa | 1 | standard | B | no | mother: renal ca, maternal aunt: breast ca, eye ca; maternal uncle: pancreatic ca | no |  |
| 31 | M | MM | 51 | IgA kappa | 1 | low | A | no | no | no |  |
| 32 | M | MM | 63 | IgA kappa | 1 | standard | A | no | no | no |  |
| 33 | F | MM | 58 | IgG kappa | 2 | high | B | no | mother: mesothelioma | no |  |
| 34 | F | MM | 63 | IgG kappa | 2 | standard | AB | no | no | no | *CEP120* c.49+485T>G |
| 35 | F | MM | 65 | IgG kappa | 2 | standard | B | no | father: gastric ca, sister: gastrointestinal ca | no | *PALB2* c.1592delT (p.Leu531CysfsTer30); *POLG* c.2209G>C (p.Gly737Arg) |
| 36 | F | MM | 67 | IgG lambda | 3 | standard | B | systemic mastocytosis, epidermoid lung cancer, essential thrombocythemia | no | no |  |
| 37 | F | MM | 59 | kappa light chain | 2 | standard | B | no | no | no |  |
| 38 | M | MM | 75 | IgG kappa | 2 | standard | CAB | no | no | no |  |
| 39 | F | MM | 56 | IgG kappa | N/A | N/A | A | N/A | N/A | yes |  |
| 40 | F | MM | 64 | IgG kappa | 3 | 0 | A | N/A | N/A | no | *BRCA2* c.9118-2A>G |
| 41 | M | MM | 63 | IgG lambda | 2 | standard | B | LGL-leukaemia, t-MDS, renal ca | N/A | no |  |
| 42 | M | MM | 58 | lambda light chain | 3 | standard | CAB | N/A | N/A | no |  |
| 43 | M | MM | 66 | IgG kappa | 2 | N/A | RA | N/A | N/A | no | *FANCG* c.1183_1192del(p.Glu395TrpfsTer5) |
| 44 | F | POEMS/MGUS | 53 | IgA lambda | N/A | N/A | N/A | basal cell ca | N/A | no | *ERCC2* c.1738G>A (p.Ala580Thr), GBA c.1226A>G (p.Asn409Ser) |
| 45 | M | MM | 54 | kappa light chain | 3 | standard | CRB | N/A | N/A | yes |  |
| 46 | M | MM | 58 | IgA kappa | N/A | N/A | A | N/A | N/A | no |  |
| 47 | M | MM | 55 | IgG kappa | 2 | standard | AB | N/A | N/A | no | *ERCC2* c.1775G>A (p.Arg592His) |
| 48 | F | MM | 68 | IgG kappa | 1 | standard | A | no | sister: breast ca | no |  |
| 49 | M | MM | 56 | IgA kappa | 1 | standard | AB | N/A | N/A | yes |  |
| 50 | M | MM | 59 | IgA lambda | 3 | standard | CRAB | no | brother: prostate ca | yes |  |
| 51 | F | MM | 51 | IgG kappa | 1 | low | A | breast ca | sister: breast ca, mother: breast ca, maternal aunt: breast ca, maternal grandmother: colon ca and lung ca, maternal grandfather: lung ca | no |  |
| 52 | F | MM | 76 | IgG kappa | 2 | high | A | no | N/A | no |  |
| 53 | M | MM | 66 | IgG kappa | 2 | standard | AB | prostate ca | N/A | no | *CHEK2* c.1229delC (p.Thr410MetfsTer15) |
| 54 | F | MM | 69 | IgA kappa | 1 | standard | B | no | sister: melanoma, sister: pancreatic ca, mother: colon ca, father: pancreatic ca, maternal aunt: colon cancer | no |  |
| 55 | M | MM | 56 | kappa light chain | 1 | standard | B | no | father: colon ca, mother: breast ca | no | *FANCM* c.5101C>T (p.Gln1701Ter) |
| 56 | M | MM | 60 | IgG kappa | 1 | standard | B | no | sister: breast ca | no |  |
| 57 | F | MM | 56 | IgG kappa | 2 | N/A | AB | N/A | N/A | no |  |
| 58 | M | MM | 57 | IgG kappa | 3 | standard | A | no | no | no |  |
| 59 | M | MM | 49 | IgG kappa | 2 | low | A | no | no | no |  |
| 60 | M | MM | 68 | IgA lambda | N/A | standard | RAB | N/A | N/A | no |  |
| 61 | M | MM | 66 | kappa light chain | 1 | standard | B | basal cell ca | father: lung ca, paternal uncle: cancer NOS, half-brother: lung cancer | no |  |
| 62 | F | MM | 68 | IgG kappa | 3 | standard | CRAB | no | N/A | no |  |
| 63 | M | MM | 61 | kappa light chain | 2 | high | RAB | N/A | N/A | no |  |
| 64 | M | MM | 67 | IgG lambda | 3 | standard | A | no | aunt: leukaemia NOS | no |  |
| 65 | M | MM | 68 | IgG kappa | 2 | standard | AB | no | father: gastric ca | no |  |
| 66 | F | MM | 73 | kappa light chain | 1 | standard | AB | no | N/A | no | *CHEK2* c.1229delC (p.Thr410MetfsTer15) |
| 67 | M | MM | 50 | kappa light chain | 1 | low | A | no | maternal aunt: ca NOS | yes |  |
| 68 | F | MM | 55 | IgA lambda | 2 | standard | AB | no | mother: breast ca, maternal aunt: breast ca, maternal cousin: breast ca, other cousin: ovarian ca, mother's father: lung ca | no |  |
| 69 | M | MM | 65 | IgG kappa | 3 | standard | RAB | N/A | N/A | no |  |
| 70 | M | MM | 63 | IgG kappa | 2 | standard | A | N/A | N/A | no |  |
| 71 | F | MM | 59 | IgG lambda | 2 | standard | 0 | no | no | no |  |
| 72 | M | MM | 71 | IgA kappa | 2 | standard | CAB | no | no | no |  |
| 73 | F | MM | 60 | IgG kappa | 2 | standard | A | no | no | no | *MUTYH* c.1187G>A (p.Gly396Asp) |
| 74 | F | MM | 58 | IgA lambda | 3 | high | CRAB | no | no | no |  |
| 75 | M | MM | 66 | non secretory | 3 | standard | RA | no | paternal uncle ca NOS, maternal cousin: ca NOS | no |  |
| 76 | F | MM | 62 | kappa light chain | 3 | standard | AB | no | no | no |  |
| 77 | M | MM | 82 | IgG kappa | 3 | standard | RAB | no | no | no |  |
| 78 | F | MM | 66 | IgA lambda | 2 | high | B | basal cell ca | father: gastric ca, sister: breast ca, brother: prostate ca | no | *CHEK2* c.1229delC (p.Thr410MetfsTer15) |
| 79 | F | MM | 54 | IgG kappa | 2 | low | AB | no | brother: cancer NOS | no | *ATM* c.7570G>C (p.Ala2524Pro) |
| 80 | M | MM | 66 | IgG kappa | 2 | standard | B | no | father: basal cell ca | no |  |
| 81 | M | MM | 66 | IgG lambda | 2 | standard | CAB | no | no | no |  |
| 82 | M | MM | 66 | IgG kappa | 2 | standard | B | osteosarcoma, T-MDS | mother: MDS - AML | no | *POT1* c.1594G>C (p.Ala532Pro) |
| 83 | M | MM | 63 | non secretory | 3 | high | RAB | no | no | no | *CTC1* c.2831delC (p.Pro944LeufsTer7) |
| 84 | F | MM | 70 | non secretory | 3 | standard | B | pancreas ca | no | no |  |
| 85 | F | MM | 54 | IgA lambda | 2 | standard | B | no | no | no |  |
| 86 | M | MM | 45 | kappa light chain | 1 | low | RB | no | Sibling AL NOS, father: lung ca, paternal aunt: lung ca | yes | *MUTYH* c.536A>G (p.Tyr179Cys) |
| 87 | F | MM | 69 | IgA lambda | 2 | standard | A | no | father: gastric ca | no |  |
| 88 | M | MM - PCL | 54 | IgG kappa | 2 | standard | AB | no | no | no | *BRCA2* c.8177A>G (p.Tyr2726Cys), *LIG4* c.2690C>G (p.Ser897Ter) |
| 89 | M | MM | 71 | IgG lambda | 2 | standard | B | rectum ca | no | no |  |
| 90 | M | MM | 59 | IgA lambda | 2 | standard | B | no | no | no |  |
| 91 | F | sMM | 61 | IgA kappa | 2 | N/A | 0 | no | maternal grandmother: leukaemia NOS | no | *CHEK2* c.1229delC (p.Thr410MetfsTer15) |
| 92 | F | MM | 67 | non secretory | 2 | standard | CB | no | mother: breast ca, father: squamous cell ca, basal cell ca | no |  |
| 93 | M | MM | 41 | IgG lambda | N/A | N/A | B | N/A | N/A | no |  |
| 94 | F | MM | 69 | non secretory | 1 | standard | B | no | no | no |  |
| 95 | M | MM - PCL | 63 | IgG kappa | 3 | standard | AB | no | mother: Burkitt lymphoma and skin ca NOS father: lung and prostate ca | no |  |
| 96 | F | MM | 75 | kappa light chain | 2 | standard | B | no | mother: pancreatic ca | no |  |
| 97 | M | MM | 56 | IgG lambda | 3 | standard | B | no | no | no | *ATR* c.516_529del (p.Val173IlefsTer7) |
| 98 | M | MM | 65 | IgA kappa | 2 | standard | 0 | no | no | no |  |
| 99 | M | MM - AL-amyloidosis | 66 | IgD lambda | 2 | standard | C | no | no | no |  |
| 100 | M | MM | 65 | IgG kappa | 2 | standard | CAB | prostate ca, basal cell ca | no | no |  |
| 101 | M | MM | 59 | IgA lambda | 3 | high | CRAB | no | no | no |  |
| 102 | M | MM | 58 | IgG kappa | 3 | standard | CB | no | sister: ALL, brother: brain ca NOS | no |  |
| 103 | F | MM | 61 | kappa light chain | 2 | high | B | no | no | no | *BLM* c.1087G>A (p.Ala363Thr) |
| 104 | F | MM | 59 | kappa light chain | 2 | standard | AB | no | no | no |  |
| 105 | F | MM | 55 | kappa light chain | 2 | standard | AB | no | mother and maternal grandfather: MM, father: esophageal ca | yes | *POT1* c.547-1G>A |
| 106 | M | MM | 51 | IgG kappa | 3 | high | AB | no | no | yes |  |
| 107 | M | MM | 62 | IgA kappa | 2 | high | AB | no | no | no |  |
| 108 | F | MM | 74 | IgG lambda | 2 | standard | 0 | no | N/A | no |  |
| 109 | F | MM | 79 | IgG kappa | 1 | standard | A | melanoma, ventricular gist -tumour | sister: breast ca, sister lung ca, brother: prostate ca | no |  |
| 110 | M | sMM | 67 | IgG lambda | 1 | standard | 0 | no | sister: lung ca, brother: colon ca | no |  |
| 111 | F | MM | 63 | IgA lambda | 3 | standard | AB | no | Father: MM, sister: skin ca NOS | no |  |
| 112 | M | MM | 73 | IgA lambda | N/A | standard | B | no | no | no |  |
| 113 | M | MM | 50 | IgA lambda | 1 | standard | A | papillary thyroidal ca | father: MM, sister: lung ca, paternal aunt: gastric ca | no | *CHEK2* c.1229delC (p.Thr410MetfsTer15), *POT1* c.458T>A (p.Leu153Ter), *MUTYH* c.1187G>A (p.Gly396Asp) |
| 114 | F | MM | 80 | IgG lambda | 3 | standard | AB | basal cell ca | no | no |  |
| 115 | M | MM | 77 | IgA kappa | 2 | high | AB | prostate ca | no | no |  |
| 116 | M | MM | 58 | IgA kappa | 3 | high | CRAB | no | father: testicular ca, mother: gastric ca | no |  |
| 117 | F | MM | 51 | IgA lambda | N/A | standard | A | N/A | N/A | no |  |
| 118 | M | MM | 63 | IgA lambda | 2 | standard | B | no | father: oral ca | no |  |
| 119 | M | MM | 67 | IgG kappa | 2 | high | B | no | no | no | *FANCM* c.5791C>T (p.Arg1931Ter) |
| 120 | M | MM | 69 | IgA lambda | 2 | high | A | prostate ca | no | no |  |
| 121 | M | MM - PCL | 60 | lambda light chain | 3 | standard | RAB | no | brother: myeloma, brother: leukaemia NOS, sister's daughter: myeloma | no |  |
| 122 | M | MM | 50 | IgG kappa | 3 | standard | A | no | no | no |  |
| 123 | M | MM | 60 | IgA lambda | 2 | high | CRAB | N/A | N/A | no |  |
| 124 | F | MM | 26 | kappa light chain | 2 | low | CAB | no | no | no |  |
| 125 | M | MM | 68 | N/A | N/A | standard | A | N/A | N/A | no |  |
| 126 | F | MM | 65 | kappa light chain | 2 | standard | B | no | maternal aunt: lymphoma NOS, maternal uncle: leukaemia NOS, maternal uncle: throat ca, maternal grandfather: prostate ca, father: prostate ca, paternal grandmother: gastric ca | no |  |
| 127 | F | MM | 77 | IgA kappa | 2 | standard | AB | no | father: gastric ca, sister: brain cancer NOS | no | *PMS2* c.765C>A (p.Tyr255Ter) |
| 128 | M | MM | 50 | IgG kappa | 1 | low | B | no | father: prostate ca | no |  |

Abbreviations: sMM: smouldering multiple myeloma ISS: The Injury Severity Score, IMWG: International Myeloma Working Group, CRAB: end-organ damage with hypercalcaemia, renal dysfunction, anaemia and bone involvement, ca: cancer, Allo-HSCT: allogeneic haematopoietic stem cell transplantation, P/LP: pathogenic / likely pathogenic, F: female, M: male, MM: multiple myeloma, N/A: not applicable, t-MDS: therapy-related myelodysplastic syndrome, CLL: chronic lymphatic leukaemia, PCL: plasma cell leukaemia, sMM: smouldering multiple myeloma, DLBCL: diffuse large B-cell lymphoma, MGUS: monoclonal gammopathy of undetermined significance, MDS: myelodysplastic syndrome, AML: acute myeloid leukaemia, AL: acute leukaemia

**Supplementary table 2: The candidate gene list**

| **Gene** | **Gene source** | **Reference** |
| --- | --- | --- |
| *AICDA* | GWAS | (7) |
| *ALP* | MM biology, bone related factor | (8) |
| *APEX1* | MM biology, DNA repair | (9) |
| *APEX2* | MM biology, DNA repair | (9) |
| *ARID1A* | Family study | (10) |
| *ASB1* | GWAS | (11) |
| *ATG5* | GWAS | (12) |
| *ATM* | MM biology, DNA repair | (9) |
| *ATR* | Family study | (13) |
| *BARD1* | MM biology, DNA repair | (14) |
| *BCL2* | MM biology, B-cell development/signalling | (15) |
| *BCL2L10* | MM biology, B-cell development/signalling | (16) |
| *BCL6* | MM biology, B-cell development/signalling | (17) |
| *BCR* | MM biology, B-cell development/signalling | (15) |
| *BLM* | MM biology, DNA repair | (9) |
| *BP1* | MM biology, DNA repair | (9) |
| *BRAF* | Somatic | (18) |
| *BRCA1* | MM biology, DNA repair | (9) |
| *BRCA2* | MM biology, DNA repair | (9) |
| *BSG* | GWAS | (19) |
| *BSP* | MM biology, bone related factor | (8) |
| *CBX7* | GWAS | (20) |
| *CCAT1* | GWAS | (12) |
| *CCDC71L* | GWAS | (20) |
| *CCND1* | Somatic / GWAS | (21) |
| *CCND3* | Somatic / GWAS | (21) |
| *CDCA7L* | GWAS | (22) |
| *CDKN2A* | Case report | (23) |
| *CDKN2C* | Somatic | (24) |
| *CEP120* | GWAS | (20) |
| *CHEK1* | MM biology, DNA repair | (9) |
| *CHEK2* | MM biology, DNA repair | (9) |
| *COL-1* | MM biology, bone related factor | (8) |
| *CRBN* | MM biology, B-cell development/signalling | (25) |
| *CTC1* | MM biology, telomere function | (26) |
| *CTLA4* | MM biology, immunogenic factor | (27) |
| *CXCR4* | MM biology, immunogenic factor | (28) |
| *CYLD* | Somatic | (29) |
| *DCHS1* | Family study | (30) |
| *DIS3* | Family study | (31) |
| *DKK1* | MM biology, bone related factor | (32) |
| *DNAH11* | GWAS | (22) |
| *DNMT3* | GWAS | (22) |
| *DTNB* | GWAS | (22) |
| *E2A* | MM biology, B-cell development/signalling | (15) |
| *EBF* | MM biology, B-cell development/signalling | (15) |
| *EBF1* | MM biology, B-cell development/signalling | (15) |
| *EGR1* | Somatic | (24) |
| *ELL2* | GWAS | (12) |
| *EP300* | Family study | (13) |
| *EPHX1* | MM biology, bone related factor | (33) |
| *ERCC1* | MM biology, DNA repair | (9) |
| *ERCC2* | MM biology, DNA repair | (9) |
| *ERCC3* | MM biology, DNA repair | (9) |
| *ERCC4* | MM biology, DNA repair | (9) |
| *ERCC5* | MM biology, DNA repair | (9) |
| *ETV6* | Case report | (34) |
| *FAM46C* | Somatic | (18) |
| *FANCA* | MM biology, DNA repair | (9) |
| *FANCB* | MM biology, DNA repair | (9) |
| *FANCC* | MM biology, DNA repair | (9) |
| *FANCD2* | MM biology, DNA repair | (9) |
| *FANCE* | MM biology, DNA repair | (9) |
| *FANCF* | MM biology, DNA repair | (9) |
| *FANCG* | MM biology, DNA repair | (9) |
| *FANCI* | MM biology, DNA repair | (9) |
| *FANCL* | MM biology, DNA repair | (9) |
| *FANCM* | MM biology, DNA repair | (9) |
| *FAT3* | Somatic | (35) |
| *FGFR3* | Somatic / GWAS | (21) |
| *FOPNL* | GWAS | (36) |
| *FOXO1* | MM biology, B-cell development/signalling | (37) |
| *GBA* | MM biology, predisposition to Gaucher disease | (38) |
| *Gsk3β* | MM biology, bone related factor | (33) |
| *HIST1H1E* | Somatic / GWAS | (21) |
| *HUWE1* | MM biology, tumour suppressor | (39) |
| *IDH1* | putative myeloma oncogene | (39) |
| *IDH2* | putative myeloma oncogene | (39) |
| *ILF2* | MM biology, DNA repair | (40) |
| *IRF4* | Somatic / GWAS | (21) |
| *JARID2* | GWAS | (12) |
| *KIF18A* | GWAS | (41) |
| *KIF1B* | Family study | (30) |
| *KLF2* | GWAS | (20) |
| *KRAS* | Somatic | (18) |
| *LIG4* | MM biology, DNA repair | (9) |
| *LSD1/KDM1A* | Family study | (42) |
| *LTB* | Somatic | (24) |
| *MAF* | Somatic / GWAS | (21) |
| *MAFB* | Somatic / GWAS | (21) |
| *MAX* | Somatic / GWAS | (21) |
| *MKL1* | Family study | (13) |
| *MLH1* | MM biology, DNA repair | (9) |
| *MMSET* | Somatic | (21) |
| *MSH2* | MM biology, DNA repair | (9) |
| *MSH6* | MM biology, DNA repair | (9) |
| *MTAP* | GWAS | (12) |
| *MUTYH* | MM biology, DNA repair | (14) |
| *MYNN* | GWAS | (43) |
| *NCOA1* | GWAS | (44) |
| *NDUFA8* | Family study | (13) |
| *NFKBIA* | Somatic | (24) |
| *NRAS* | Somatic | (18) |
| *OCN* | MM biology, osteogenic differentiation | (8) |
| *OGG1* | MM biology, DNA repair | (14) |
| *OPN* | MM biology, osteogenic differentiation | (8) |
| *OSX* | MM biology, osteogenic differentiation | (8) |
| *PALB2* | MM biology, DNA repair | (45) |
| *PARP1* | MM biology, DNA repair | (14) |
| *PARPP* | MM biology, DNA repair | (9) |
| *PAX5* | MM biology, B-cell development/signalling | (15) |
| *PCNA* | MM biology, DNA repair | (9) |
| *PDPK1* | Family study | (13) |
| *PMS1* | MM biology, DNA repair | (9) |
| *PMS2* | MM biology, DNA repair | (9) |
| *POT1* | GWAS | (20) |
| *POUF51* | GWAS | (43) |
| *PRDK2* | Somatic / GWAS | (21) |
| *PRDM1* | Somatic | (46) |
| *PREX1* | GWAS | (47) |
| *PRKD2* | putative myeloma oncogene | (39) |
| *PRR14* | GWAS | (20) |
| *PSAP* | MM biology, predisposition to Gaucher disease | (38) |
| *PSORS1C2* | GWAS | (43) |
| *PTPN11* | putative myeloma oncogene | (39) |
| *RAD50* | MM biology, DNA repair | (9) |
| *RAD51* | MM biology, DNA repair | (14) |
| *RAG1* | MM biology, B-cell development/signalling | (37) |
| *RAG2* | MM biology, B-cell development/signalling | (37) |
| *RB1* | Somatic | (29) |
| *RECQ1* | GWAS | (48) |
| *RFWD3* | GWAS | (12) |
| *ROBO1* | Somatic | (24) |
| *RPN11* | MM biology, DNA repair | (49) |
| *RUNX2* | MM biology, osteogenic differentiation | (8) |
| *SETBP1* | GWAS | (47) |
| *SF3B1* | putative myeloma oncogene | (39) |
| *SIRT6* | MM biology, DNA repair | (50) |
| *SMARCD3* | GWAS | (12) |
| *SOHLH2* | GWAS | (51) |
| *SOST* | MM biology, bone related factor | (32) |
| *SP140* | Somatic | (24) |
| *SP3* | GWAS | (20) |
| *STK4* | MM biology, immunogenic factor | (11) |
| *TAZ* | Somatic | (52) |
| *TERC* | GWAS | (43) |
| *THPO* | Case report | (53) |
| *TIE2* | MM biology, angiogenesis | (54) |
| *TNFRSF13B* | GWAS | (43) |
| *TP53* | Somatic | (35) |
| *TRAF3* | Somatic | (35) |
| *ULK4* | GWAS | (22) |
| *URB5* | MM biology, tumour suppressor | (39) |
| *USP1* | MM biology, DNA repair | (55) |
| *USP45* | Family study | (10) |
| *VEGF* | MM biology, angiogenesis | (56) |
| *VEGFR2* | MM biology, angiogenesis | (56) |
| *WAC* | GWAS | (11) |
| *WWOX* | Somatic | (57) |
| *XPC* | MM biology, DNA repair | (9) |
| *XRCC3* | MM biology, DNA repair | (58) |
| *YAP1* | MM biology, DNA repair | (59) |

**Supplementary table 3: Pathogenic and likely pathogenic variants of dominant and recessively inherited genes**

| **ID** | **Gene** | **Inheritance** | **Variant** | **OR (95% CI) compared to non-cancer gnomAD global** | **OR (95% CI) compared to non-cancer gnomAD Finns** | **AF gnomAD exomes global / Finnish** | **Coding impact** | **ACMG/AMP Classification (stratification criteria fulfilled)** | **DG** | **Age at DG** | **Gender** | **Paraprotein subtype** | **Other primary malignancies** | **Family history of cancer (ca) or haematological disease** |
| --- | --- | --- | --- | --- | --- | --- | --- | --- | --- | --- | --- | --- | --- | --- |
| 79 | ***ATM*** | **AD** | (NM_000051.4):c.7570G>C (p.Ala2524Pro) | 154 (18-1289) | 14 (2-118) | 0.00003 / 0.00028 | missense | LP(PM1, PM2, PP5, PP3) | MM | 54 | F | IgG kappa | no | brother: cancer NOS |
| 97 | ***ATR*** | **AD** | (NM_001184.4):c.516_529del (p.Val173IlefsTer7) | 455 (41-5056) | N/A | 0.00002 / 0.00019 | frameshift | P (PVS1, PM2, PP3 ) | MM | 56 | M | IgG lambda | no | no |
| 40 | ***BRCA2*** | **AD** | (NM_000059.4):c.9118-2A>G | N/A | N/A | 0.00003 / 0.00037 | missense | P (PP5, PM1, PM2, PP3) | MM | 64 | F | IgG kappa | N/A | N/A |
| 88 | ***BRCA2*** | **AD** | (NM_000059.4):c.8177A>G (p.Tyr2726Cys) | 115 (14-928) | 11 (1.3-85) | N/A / N/A | missense | P (PVS1, PM1, PM2) | MM - PCL | 54 | M | IgG kappa | no | no |
|  | *LIG4* | AR | (NM_206937.2):c.2690C>G (p.Ser897Ter) | 992 (57-14835) | N/A | 0.000004 / N/A | missense | P (PVS1, PM2) |  |  |  |  |  |  |
| 53 | ***CHEK2*** | **AD** | (NM_001005735.2):c.1229delC (p.Thr410MetfsTer15) | 10 (4-24) | 2 (0.9 - 5.6) | 0.00205 / 0.00874 | frameshift | P (PVS1, PM2, PP5) | MM | 66 | M | IgG kappa | prostate ca | N/A |
| 66 | ***CHEK2*** | **AD** | (NM_001005735.2):c.1229delC (p.Thr410MetfsTer15) | 10 (4-24) | 2 (0.9 - 5.6) | 0.00205 / 0.00874 | frameshift | P (PVS1, PM2, PP5) | MM | 73 | F | kappa light chain | no | N/A |
| 78 | ***CHEK2*** | **AD** | (NM_001005735.2):c.1229delC (p.Thr410MetfsTer15) | 10 (4-24) | 2 (0.9 - 5.6) | 0.00205 / 0.00874 | frameshift | P (PVS1, PM2, PP5) | MM | 66 | F | IgA lambda | basal-cell ca | father: gastric ca (died at 43), sister: breast ca, brother: prostate ca |
| 91 | ***CHEK2*** | **AD** | (NM_001005735.2):c.1229delC (p.Thr410MetfsTer15) | 10 (4-24) | 2 (0.9 - 5.6) | 0.00205 / 0.00874 | frameshift | P (PVS1, PM2, PP5) | sMM | 61 | F | IgA kappa | no | mother's mother: leukaemia NOS |
| 113 | ***CHEK2*** | **AD** | (NM_001005735.2):c.1229delC (p.Thr410MetfsTer15) | 10 (4-24) | 2 (0.9 - 5.6) | 0.00205 / 0.00874 | frameshift | P (PVS1, PM2, PP5) | MM | 50 | M | IgA lambda | papillary thyroid ca (diagnosed at 36) | father: MM (diagnosed at 76), sister: lung ca, paternal aunt: gastric ca |
|  | ***POT1*** | **AD** | (NM_015450.3):c.458T>A (p.Leu153Ter) | N/A | N/A | N/A / N/A | nonsense | P (PVS1, PM2, PP3) |  |  |  |  |  |  |
|  | *MUTYH* | AR | (NM_001128425.2):c.1187G>A (p.Gly396Asp) | 3 (0.7-11) | 4 (0.9-15) | 0.00295 / 0.0022 | missense | P (PP5, PS3, PP3, PM5) |  |  |  |  |  |  |
| 82 | ***POT1*** | **AD** | (NM_015450.3):c.1594G>C (p.Ala532Pro) | 913 (57-14686) | N/A | 0.00003/ N/A | missense | LP (PP5, PS1, PM2, PP3) | MM - t-MDS | 66 | M | IgG kappa | osteosarcoma, T-MDS | mother: AML |
| 105 | ***POT1*** | **AD** | (NM_015450.3):c.547-1G>A | N/A | N/A | N/A / N/A | splicing | P (PVS1, PM2, PP3) | MM | 55 | F | kappa light chain | no | mother: MM, mother's father: MM, father: esophageal ca |
| 35 | ***PALB2*** | **AD** | (NM_024675.4):c.1592delT (p.Leu531CysfsTer30) | 20 (3-147) | 1.9 (0.3-14) | 0.00019 / 0.00203 | frameshift | P (PVS1, PM2) | MM | 65 | F | IgG kappa | no | father: gastric ca, sister: gastrointestinal ca (died at 41) |
| 105 | *BLM* | AR | (NM_000057.4):c.1087G>A (p.Ala363Thr) | 46 (6-345) | 5 (0.6-35) | 0.00008 / 0.00084 | missense | LP (PP3, PM2) | MM | 61 | F | kappa light chain | no | no |
| 34 | *CEP120* | AR | (NM_153223.3):c.49+485T>G | 125 (14-1127) | 10 (0.9-116) | 0.00003 / 0.00037 | splicing | LP (PVS1, PM2) | MM | 63 | F | IgG kappa | no | no |
| 83 | *CTC1* | AR | (NM_025099.6):c.2831delC (p.Pro944LeufsTer7) | 9 (1-67) | 1.4 (0.2-11) | 0.00042 / 0.00269 | frameshift | P (PVS1, PP5, PM2) | MM | 63 | M | non secretory | no | no |
| 47 | *ERCC2* | AR | (NM_000400.4):c.1775G>A (p.Arg592His) | 14 (2-103) | 6 (0.8-46) | 0.00002 / N/A | missense | LP (PM1, PM2, PM5, PP3) | MM | 55 | M | IgG kappa | N/A | N/A |
| 44 | *ERCC2* | AR | (NM_000400.4):c.1738G>A (p.Ala580Thr) | 231 (26-2083) | N/A | 0.00028 / 0.00065 | missense | LP (PM1, PM2, PP3) | MGUS and POEMS | 53 | F | IgA lambda and IgG kappa | basal-cell ca | N/A |
|  | *GBA* | AR | (NM_001005742.3):c.1226A>G (p.Asn409Ser) | 2 (0.2-12) | 3 (0.5-25) | 0.00229 / 0.00116 | missense | P (PP5, PS3, PM1, PM5, PP2, PP3) |  |  |  |  |  |  |
| 43 | *FANCG* | AR | (NM_004629.2):c.1183_1192del(p.Glu395TrpfsTer5) | 75 (10-590) | 38 (3-426) | 0.00005 / 0.00010 | frameshift | P (PVS1, PM2, PP5) | MM | 66 | M | IgG kappa | N/A | N/A |
| 24 | *FANCI* | AR | (NM_001113378.2):c.286G>A (p.Glu96Lys) | 2 (0.3-16) | 2 (0.3-15) | 0.00178 / 0.00185 | missense | LP (PP3, PM2, BP6) | MM- PCL | 69 | F | IgG kappa | no | no |
| 5 | *FANCM* | AR | (NM_020937.4):c.5101C>T (p.Gln1701Ter) | 6 (2-25) | 0.8 (0.1-6) | 0.00129 / 0.00823 | nonsense | P (PVS1, PP5, PM2) | MM | 66 | M | IgG lambda | no | no |
| 55 | *FANCM* | AR | (NM_020937.4):c.5101C>T (p.Gln1701Ter) | 6 (2-25) | 0.8 (0.1-6) | 0.00129 / 0.00823 | nonsense | P (PVS1, PP5, PM2) | MM | 56 | M | kappa light chain | no | father: colon ca, mother: breast ca |
| 122 | *FANCM* | AR | (NM_020937.4):c.5791C>T (p.Arg1931Ter) | 4 (0.5-28) | 3 (0.4-25) | 0.00101 / 0.00448 | nonsense | P (PVS1, PM2) | MM | 67 | M | IgG kappa | no | no |
| 73 | *MUTYH* | AR | (NM_001128425.2):c.1187G>A (p.Gly396Asp) | 3 (0.7-11) | 4 (0.9-15) | 0.00295 / 0.0022 | missense | P (PP5, PS3, PP3, PM5) | MM | 60 | F | IgG kappa | no | no |
| 21 | *MUTYH* | AR | (NM_001128425.2):c.536A>G (p.Tyr179Cys) | 5 (1.3-21) | 5 (1.2-22) | 0.00154 / 0.00153 | missense | P (PP5, PM1, PM2, PP2, PP3) | MM | 45 | M | IgG kappa | no | N/A |
| 86 | *MUTYH* | AR | (NM_001128425.2):c.536A>G (p.Tyr179Cys) | 5 (1.3-21) | 5 (1.2-22) | 0.00154 / 0.00153 | missense | P (PP5, PM1, PM2, PP2, PP3) | MM | 45 | M | kappa light chain | no | sibling: acute leukaemia, father: lung ca, paternal aunt: lung ca |
| 130 | *PMS2* | AR | (NM_000535.7):c.765C>A (p.Tyr255Ter) | N/A | N/A | N/A / N/A | nonsense | P (PVS1, PP5, PM2) | MM | 77 | F | IgA kappa | no | father: gastric ca, sister: brain ca (died at 51) |
| 18 | *RAG1* | AR | (NM_000448.3):c.2615delT (p.Leu872Ter) | 924 (57-14847) | 84 (5-1353) | 0.000004 / 0.00005 | frameshift | P (PVS1, PM2) | MM | 56 | F | lambda light chain | breast ca | 2 sisters: breast ca |

Abbreviations: OR: odds ratio, CI: confidence interval, AF: allele frequency, gnomAD: Genome Aggregation Database, ACMG/AMP: American College of Medical Genetics and Genomics/Association for Molecular Pathology (stratification criteria fulfilled are in order of the strongest pathogenic criteria first), DG: diagnosis, AD: autosomal dominant, LP: likely pathogenic, PM1-2: moderate evidence of pathogenicity, PP2,3,5: supporting evidence of pathogenicity, PVS1: very strong evidence of pathogenicity, PS3: strong evidence of pathogenicity,  BP4: strong evidence of benign impact, MM: multiple myeloma, NOS: not otherwise specified, N/A: not applicable, AR: autosomal recessive, P: pathogenic, PCL: plasma cell leukaemia, sMM: smouldering myeloma, t-MDS: therapy related myelodysplastic syndrome, MGUS: monoclonal gammopathy of undetermined significance.

**Supplementary references**

1. Wartiovaara-Kautto U, Hirvonen EAM, Pitkanen E, Heckman C, Saarela J, Kettunen K, et al. Germline alterations in a consecutive series of acute myeloid leukemia. Leukemia. 2018;32(10):2282-5.

2. Katainen R, Donner I, Cajuso T, Kaasinen E, Palin K, Mäkinen V, et al. BasePlayer: Versatile Analysis Software for Large-scale Genomic Variant Discovery. bioRxiv. 2017:126482.

3. Karczewski KJ, Francioli LC, Tiao G, Cummings BB, Alföldi J, Wang Q, et al. The mutational constraint spectrum quantified from variation in 141,456 humans. Nature. 2020;581(7809):434-43.

4. Christos Kopanos VT, Alexandros Kouris, Charles E Chapple, Monica Albarca Aguilera, Richard Meyer, Andreas Massouras. VarSome: the human genomic variant search engine. Bioinformatics. 1 June 2019;35(11):1978–80.

5. Tate JG, Bamford S, Jubb HC, Sondka Z, Beare DM, Bindal N, et al. COSMIC: the Catalogue Of Somatic Mutations In Cancer. Nucleic Acids Research. 2018;47(D1):D941-D7.

6. R Development Core Team. R: A language and environment for statistical computing. 4.1.0 ed: R Foundation for Statsistical Computing. Retrieved from <http://www.R-project.org>.; 2010.

7. Campa D, Martino A, Macauda A, Dudziński M, Suska A, Druzd-Sitek A, et al. Genetic polymorphisms in genes of class switch recombination and multiple myeloma risk and survival: an IMMEnSE study. Leuk Lymphoma. 2019;60(7):1803-11.

8. Guo J, Fei C, Zhao Y, Zhao S, Zheng Q, Su J, et al. Lenalidomide restores the osteogenic differentiation of bone marrow mesenchymal stem cells from multiple myeloma patients via deactivating Notch signaling pathway. Oncotarget. 2017;8(33):55405.

9. Kassambara A, Gourzones-Dmitriev C, Sahota S, Rème T, Moreaux J, Goldschmidt H, et al. A DNA repair pathway score predicts survival in human multiple myeloma: the potential for therapeutic strategy. Oncotarget. 2014;5(9):2487-98.

10. Waller RG, Darlington TM, Wei X, Madsen MJ, Thomas A, Curtin K, et al. Novel pedigree analysis implicates DNA repair and chromatin remodeling in multiple myeloma risk. PLoS Genet. 2018;14(2):e1007111.

11. Janz S, Zhan F, Sun F, Cheng Y, Pisano M, Yang Y, et al. Germline Risk Contribution to Genomic Instability in Multiple Myeloma. Front Genet. 2019;10:424.

12. Mitchell JS, Li N, Weinhold N, Försti A, Ali M, van Duin M, et al. Genome-wide association study identifies multiple susceptibility loci for multiple myeloma. Nat Commun. 2016;7:12050.

13. Bolli N, Barcella M, Salvi E, D'Avila F, Vendramin A, De Philippis C, et al. Next-generation sequencing of a family with a high penetrance of monoclonal gammopathies for the identification of candidate risk alleles. Cancer. 2017;123(19):3701-8.

14. Gourzones-Dmitriev C, Kassambara A, Sahota S, Rème T, Moreaux J, Bourquard P, et al. DNA repair pathways in human multiple myeloma: role in oncogenesis and potential targets for treatment. Cell Cycle. 2013;12(17):2760-73.

15. Siitonen TK, P. Lymfaattisten solujen tuotanto. In: Porkka KL, R.; Remes, K.; Savolainen, E-R, editor. Veritaudit. 4 ed. Helsinki: Kustannus Oy Duodecim; 2015. p. 27-30.

16. Hamouda MA, Jacquel A, Robert G, Puissant A, Richez V, Cassel R, et al. BCL-B (BCL2L10) is overexpressed in patients suffering from multiple myeloma (MM) and drives an MM-like disease in transgenic mice. J Exp Med. 2016;213(9):1705-22.

17. Tahara K, Takizawa M, Yamane A, Osaki Y, Ishizaki T, Mitsui T, et al. Overexpression of B-cell lymphoma 6 alters gene expression profile in a myeloma cell line and is associated with decreased DNA damage response. Cancer Sci. 2017;108(8):1556-64.

18. Chapman MA, Lawrence MS, Keats JJ, Cibulskis K, Sougnez C, Schinzel AC, et al. Initial genome sequencing and analysis of multiple myeloma. Nature. 2011;471(7339):467-72.

19. Łacina P, Butrym A, Mazur G, Bogunia-Kubik K. BSG and MCT1 Genetic Variants Influence Survival in Multiple Myeloma Patients. Genes (Basel). 2018;9(5).

20. Went M, Sud A, Försti A, Halvarsson B-M, Weinhold N, Kimber S, et al. Identification of multiple risk loci and regulatory mechanisms influencing susceptibility to multiple myeloma. Nature Communications. 2018;9(1):3707.

21. Walker BA, Boyle EM, Wardell CP, Murison A, Begum DB, Dahir NM, et al. Mutational Spectrum, Copy Number Changes, and Outcome: Results of a Sequencing Study of Patients With Newly Diagnosed Myeloma. J Clin Oncol. 2015;33(33):3911-20.

22. Broderick P, Chubb D, Johnson DC, Weinhold N, Försti A, Lloyd A, et al. Common variation at 3p22. 1 and 7p15. 3 influences multiple myeloma risk. Nature genetics. 2012;44(1):58-61.

23. Shah V, Boyd KD, Houlston RS, Kaiser MF. Constitutional mutation in CDKN2A is associated with long term survivorship in multiple myeloma: a case report. BMC Cancer. 2017;17(1):718-.

24. Bolli N, Avet-Loiseau H, Wedge DC, Van Loo P, Alexandrov LB, Martincorena I, et al. Heterogeneity of genomic evolution and mutational profiles in multiple myeloma. Nat Commun. 2014;5:2997.

25. Butrym A, Łacina P, Rybka J, Chaszczewska-Markowska M, Mazur G, Bogunia-Kubik K. Cereblon and IRF4 variants affect risk and response to treatment in multiple myeloma. Archivum immunologiae et therapiae experimentalis. 2016;64(1):151-6.

26. Hyatt S, Jones RE, Heppel NH, Grimstead JW, Fegan C, Jackson GH, et al. Telomere length is a critical determinant for survival in multiple myeloma. Br J Haematol. 2017;178(1):94-8.

27. Egg D, Schwab C, Gabrysch A, Arkwright PD, Cheesman E, Giulino-Roth L, et al. Increased Risk for Malignancies in 131 Affected CTLA4 Mutation Carriers. Frontiers in Immunology. 2018;9(2012).

28. Ullah TR. The role of CXCR4 in multiple myeloma: Cells' journey from bone marrow to beyond. J Bone Oncol. 2019;17:100253-.

29. Mikulasova A, Wardell CP, Murison A, Boyle EM, Jackson GH, Smetana J, et al. The spectrum of somatic mutations in monoclonal gammopathy of undetermined significance indicates a less complex genomic landscape than that in multiple myeloma. Haematologica. 2017;102(9):1617-25.

30. Catalano C, Paramasivam N, Blocka J, Giangiobbe S, Huhn S, Schlesner M, et al. Characterization of rare germline variants in familial multiple myeloma. Blood Cancer J. 2021;11(2):33.

31. Pertesi M, Vallée M, Wei X, Revuelta MV, Galia P, Demangel D, et al. Exome sequencing identifies germline variants in DIS3 in familial multiple myeloma. Leukemia. 2019;33(9):2324-30.

32. Terpos E, Ntanasis-Stathopoulos I, Gavriatopoulou M, Dimopoulos MA. Pathogenesis of bone disease in multiple myeloma: from bench to bedside. Blood Cancer J. 2018;8(1):7.

33. Durie B, Van Ness B, Ramos C, Stephens O, Haznadar M, Hoering A, et al. Genetic polymorphisms of EPHX1, Gsk3β, TNFSF8 and myeloma cell DKK-1 expression linked to bone disease in myeloma. Leukemia. 2009;23(10):1913-9.

34. Zhang MY, Churpek JE, Keel SB, Walsh T, Lee MK, Loeb KR, et al. Germline ETV6 mutations in familial thrombocytopenia and hematologic malignancy. Nature Genetics. 2015;47(2):180-5.

35. Walker BA, Wardell CP, Murison A, Boyle EM, Begum DB, Dahir NM, et al. APOBEC family mutational signatures are associated with poor prognosis translocations in multiple myeloma. Nat Commun. 2015;6:6997.

36. Ziv E, Dean E, Hu D, Martino A, Serie D, Curtin K, et al. Genome-wide association study identifies variants at 16p13 associated with survival in multiple myeloma patients. Nature Communications. 2015;6(1):7539.

37. Szydłowski M, Jabłońska E, Juszczyński P. FOXO1 transcription factor: a critical effector of the PI3K-AKT axis in B-cell development. International reviews of immunology. 2014;33(2):146-57.

38. Weinreb NJ, Mistry PK, Rosenbloom BE, Dhodapkar MV. MGUS, lymphoplasmacytic malignancies, and Gaucher disease: the significance of the clinical association. Blood. 2018;131(22):2500-1.

39. Walker BA, Mavrommatis K, Wardell CP, Ashby TC, Bauer M, Davies F, et al. A high-risk, Double-Hit, group of newly diagnosed myeloma identified by genomic analysis. Leukemia. 2019;33(1):159-70.

40. Marchesini M, Ogoti Y, Fiorini E, Aktas Samur A, Nezi L, D'Anca M, et al. ILF2 Is a Regulator of RNA Splicing and DNA Damage Response in 1q21-Amplified Multiple Myeloma. Cancer Cell. 2017;32(1):88-100.e6.

41. Scales M, Chubb D, Dobbins SE, Johnson DC, Li N, Sternberg MJ, et al. Search for rare protein altering variants influencing susceptibility to multiple myeloma. Oncotarget. 2017;8(22):36203-10.

42. Wei X, Calvo-Vidal MN, Chen S, Wu G, Revuelta MV, Sun J, et al. Germline lysine-specific demethylase 1 (LSD1/KDM1A) mutations confer susceptibility to multiple myeloma. Cancer research. 2018;78(10):2747-59.

43. Chubb D, Weinhold N, Broderick P, Chen B, Johnson DC, Försti A, et al. Common variation at 3q26. 2, 6p21. 33, 17p11. 2 and 22q13. 1 influences multiple myeloma risk. Nature genetics. 2013;45(10):1221-5.

44. Peng M, Zhao G, Yang F, Cheng G, Huang J, Qin X, et al. NCOA1 is a novel susceptibility gene for multiple myeloma in the Chinese population: A case-control study. PloS one. 2017;12(3):e0173298.

45. Pawlyn C, Loehr A, Ashby C, Tytarenko R, Deshpande S, Sun J, et al. Loss of heterozygosity as a marker of homologous repair deficiency in multiple myeloma: a role for PARP inhibition? Leukemia. 2018;32(7):1561-6.

46. Bolli N, Biancon G, Moarii M, Gimondi S, Li Y, de Philippis C, et al. Analysis of the genomic landscape of multiple myeloma highlights novel prognostic markers and disease subgroups. Leukemia. 2018;32(12):2604-16.

47. Chattopadhyay S, Thomsen H, da Silva Filho MI, Weinhold N, Hoffmann P, Nöthen MM, et al. Enrichment of B cell receptor signaling and epidermal growth factor receptor pathways in monoclonal gammopathy of undetermined significance: a genome-wide genetic interaction study. Molecular Medicine. 2018;24(1):30.

48. Viziteu E, Klein B, Basbous J, Lin YL, Hirtz C, Gourzones C, et al. RECQ1 helicase is involved in replication stress survival and drug resistance in multiple myeloma. Leukemia. 2017;31(10):2104-13.

49. Song Y, Li S, Ray A, Das DS, Qi J, Samur MK, et al. Blockade of deubiquitylating enzyme Rpn11 triggers apoptosis in multiple myeloma cells and overcomes bortezomib resistance. Oncogene. 2017;36(40):5631-8.

50. Cea M, Cagnetta A, Adamia S, Acharya C, Tai YT, Fulciniti M, et al. Evidence for a role of the histone deacetylase SIRT6 in DNA damage response of multiple myeloma cells. Blood. 2016;127(9):1138-50.

51. Duran-Lozano L, Thorleifsson G, Lopez de Lapuente Portilla A, Niroula A, Went M, Thodberg M, et al. Germline variants at SOHLH2 influence multiple myeloma risk. Blood Cancer J. 2021;11(4):76.

52. Grieve S, Wajnberg G, Lees M, Chacko S, Weir J, Crapoulet N, et al. TAZ functions as a tumor suppressor in multiple myeloma by downregulating MYC. Blood advances. 2019;3(22):3613-25.

53. Stockklausner C, Echner N, Klotter A-C, Hegenbart U, Dreger P, Kulozik AE. Hereditary thrombocythemia caused by a thrombopoietin (THPO) gain-of-function mutation associated with multiple myeloma and congenital limb defects. Annals of hematology. 2012;91(7):1129-33.

54. Huang H, Bhat A, Woodnutt G, Lappe R. Targeting the ANGPT–TIE2 pathway in malignancy. Nature Reviews Cancer. 2010;10(8):575-85.

55. Das DS, Das A, Ray A, Song Y, Samur MK, Munshi NC, et al. Blockade of Deubiquitylating Enzyme USP1 Inhibits DNA Repair and Triggers Apoptosis in Multiple Myeloma Cells. Clin Cancer Res. 2017;23(15):4280-9.

56. Sissung TM, Peer CJ, Korde N, Mailankody S, Kazandjian D, Venzon DJ, et al. Carfilzomib and lenalidomide response related to VEGF and VEGFR2 germline polymorphisms. Cancer chemotherapy and pharmacology. 2017;80(1):217-21.

57. Hussain T, Liu B, Shrock MS, Williams T, Aldaz CM. WWOX, the FRA16D gene: A target of and a contributor to genomic instability. Genes Chromosomes Cancer. 2019;58(5):324-38.

58. Vangsted A, Gimsing P, Klausen TW, Nexø BA, Wallin H, Andersen P, et al. Polymorphisms in the genes ERCC2, XRCC3 and CD3EAP influence treatment outcome in multiple myeloma patients undergoing autologous bone marrow transplantation. International Journal of Cancer. 2007;120(5):1036-45.

59. Cottini F, Hideshima T, Xu C, Sattler M, Dori M, Agnelli L, et al. Rescue of Hippo coactivator YAP1 triggers DNA damage–induced apoptosis in hematological cancers. Nature medicine. 2014;20(6):599-606.
